# Better off alone? Compared performance of monoclonal and polyclonal stands of a cultivated red alga growth

**DOI:** 10.1111/eva.12908

**Published:** 2020-01-22

**Authors:** Sara Usandizaga, Alejandro H. Buschmann, Carolina Camus, José Luis Kappes, Sophie Arnaud‐Haond, Stéphane Mauger, Myriam Valero, Marie Laure Guillemin

**Affiliations:** ^1^ Programa de Doctorado en Ciencias, mención Conservación y Manejo de Recursos Naturales Centro i~mar and CeBiB Universidad de Los Lagos Puerto Montt Chile; ^2^ Centro i~mar and CeBiB Universidad de Los Lagos Puerto Montt Chile; ^3^ Laboratoire Environnement Profond‐Centre de Brest IFREMER Plouzané France; ^4^ CNRS Sorbonne Université UMI 3614 Evolutionary Biology and Ecology of Algae Universidad Austral de Chile Roscoff cedex France; ^5^ CNRS Sorbonne Université UMI 3614 Evolutionary Biology and Ecology of Algae Pontificia Universidad Católica de Chile Universidad Austral de Chile Roscoff cedex France; ^6^ Facultad de Ciencias Centro FONDAP de Investigación en Dinámica de Ecosistemas Marinos de Altas Latitudes (IDEAL) Instituto de Ciencias Ambientales y Evolutivas Universidad Austral de Chile Valdivia Chile

**Keywords:** domestication, general‐purpose genotypes, genotypic diversity, productivity, seaweed

## Abstract

The objective of this study was to test, using a field experiment, the effect of genotypic diversity on productivity of farmed populations (Ancud and Chaica, Chile) of the domesticated red alga *Agarophyton chilense* (formerly known as *Gracilaria chilensis*), a species considered as economically important in Chile. Monoclonal and polyclonal (4 and 8 genotypes) subplots were outplanted into the mid intertidal in Metri Bay (Puerto Montt, Chile) during summer, a season in which *A. chilense* face higher temperatures (>18°C) and low nitrogen availability (<4.00 μmol). Ancud farm genotypes show higher growth rates in the monoclonal rather than the two polyclonal subplots. A similar tendency, yet not significant, was discernible in Chaica. In addition, whatever the population of origin of the thalli, no effect of genotypic diversity was detected neither on the agar yield and its quality, nor on the epiphyte load. Such unexpected results of a higher performance in plots with a lower genotypic diversity could be explained (a) by human‐assisted selection for dominant‐best‐performing genotypes that could counterbalance the negative effect caused by the low genotypic diversity in farms and (b) by the fact that the organisms inhabiting the algal mats do not impact the fitness of their host. Overall, the results obtained here suggest that despite farm induced selection lead to impoverished pools of genotypes, they may also have a positive effect of on the resistance of farmed populations to seasonal stressors. However, whether this may have a secondary negative effect on the longer term in a fluctuating environment remains to be determined, but may be avoided by adopting strategy of selection favoring different genotypes in space and time, as implemented in forestry.

## 1 INTRODUCTION

Intraspecific genetic diversity is a key factor to guide restoration programs for population resilience and resistance to anthropogenic stressors in obligatory sexual reproducing species (Johnson, Martin, Cairney, & Anderson, 2012). It is widely assumed that reduction in population size, such as promoted by strong bottlenecks, can lead to rapid loss of genetic variation potentially leading to a decrease of the “evolutionary potential” of a population and to an accumulation of deleterious mutations (Arnaud‐Haond, Marbà, Diaz‐Almela, Serrão, & Duarte, 2010). Indeed, several genetic rescue studies have demonstrated that introducing new genetic diversity in restoration programs could enhance a population's adaptive potential (DeWald & Kolanoski, 2017). However, this is not always the case and the reverse effect can be observed as a result of outbreeding depression when recombination with the new introduced genotypes leads to the disruption of adapted gene complexes and consequently to suboptimal adaptation (Edmands, 2007; Olivieri, Tonnabel, Ronce, & Mignot, 2016). Predictions are yet more difficult to make for partially clonal species for which clonal growth of fit and complementary genotypes may partially compensate for the negative effects of outbreeding depression resulting from some sexual crosses (Lynch, 1984).

In partially clonal species, most experiments testing the impact of genetic diversity on fitness used, as a *proxy*, genotypic diversity (i.e., number of genotypes arising from distinct events of sexual reproduction observed at the population level). Genotypic diversity can influence a population's demographic responses (shoot density: Hughes & Stachowicz, 2004, leaf shoots: Reusch, Ehlers, Haemmerli, & Worm, 2005; shoot density, mortality, recruitment, and net population growth rate: Arnaud‐Haond et al., 2010). Several empirical studies in partially clonal organisms have also shown that higher genotypic diversity was related to higher resilience (seagrasses: Ehlers, Worm, & Reusch, 2008; Reusch et al., 2005), higher resistance to disturbance and invasion (terrestrial plants: Booth & Grime, 2003, Crutsinger, Souza, & Sanders, 2008, Prieto et al., 2015; seagrasses: Hughes & Stachowicz, 2004), and lower parasite prevalence (animals: Altermatt & Ebert, 2008; King, Jokela, & Lively, 2011). In the same way, numerous studies highlight the importance of genotypic diversity in domesticated species, indicating that domestication has led to a reduction in genotypic diversity for several asexually cultivated plants (Glendinning, 1983) and seaweeds (Guillemin et al., 2008; Huh, Lee, Lee, & Choi, 2004; Valero et al., 2017). It has been proposed that highly reduced genetic and/or genotypic diversity could lead to higher susceptibility to epiphytes or disease in crops (potatoes: Provan et al., 1999; rice: Zhu et al., 2000; *Kappaphycus*: Hurtado, Neish, & Critchley, 2015) and could be, in part, contributory to the recent decline in Chilean macroalgal farms biomass production (*Agarophyton chilense* landing: Servicio nacional de pesca y acuicultura, 2015; this species was previously referred as *Gracilaria chilensis* see Gurgel, Norris, Schmidt, Le, & Fredericq, 2018). As also reported in plants and seagrasses, one study undertaken in the invasive red alga *Agarophyton vermiculophyllum*, showed a general positive effect of genotypic diversity on growth and productivity (as *Gracilaria vermiculophylla*, Gerstenmeier, Krueger‐Hadfield, & Sotka, 2016). Despite this apparent agreement between studies on clonal organisms, rate of genotypes turnover and lifespan of clones can strongly impact the effect of genotypic diversity on populations.

Indeed, in long‐lived clonal species such as *Zostera marina, Zostera noltii,* and *Posidonia oceanica* some results contrasting with the main hypothesis of positive effect of genotypic diversity on populations demographic responses have been reported (Díaz‐Almela et al., 2007; Massa, Paulino, Serrao, Duarte, & Arnaud‐Haond, 2013). For example, in *P. oceanica*, high genetic diversity has been related to enhanced survival of plants in transplants experiments (Procaccini & Piazzi, 2001), while lower mortality rates have been reported in natural meadows subjected to aquaculture impacts and characterized by very low genotypic and genetic diversity when compared to more diverse ones (Diaz‐Almela et al., 2007). Meadows of these seagrass species are characterized by the existence of genotypes of very distinct clonal size with highly dominant clones scaling up to tens of thousands of plants mixed with very small clones for which only a very small number of plants are encountered (Arnaud‐Haond et al., 2010; Reusch et al., 2005), and various hypotheses have been advanced to explain these results. First, large clones could be very old and thrive in distinct habitats (the bigger clones are distributed over whole biogeographic region) that have changed through time (the bigger clones seem to be established since the Pleistocene in *P. oceanica*; Arnaud‐Haond et al., 2012). These large and old genotypes could be highly fit and adapted to a wide range of environmental conditions (Diaz‐Almela et al., 2007), following the “general‐purpose genotype” hypothesis (Baker, 1965 in Lynch, 1984; Vrijenhoek & Parker, 2009). Populations dominated by these large clones could be more resistant to perturbations than more diverse ones consisting of many smaller and younger clones, counterbalancing the positive effect of the genotypic diversity (Diaz‐Almela et al., 2007; Reusch & Hughes, 2006). If the settlement of new seeds (i.e., new genotypes coming from sexual reproduction) in the meadows is uncommon, genotypic diversity will steadily decline at each generation after population establishment since the dominant‐best‐performing genotypes survive and reproduce more and the positive effects of genotypic diversity could be short‐lived (Aguirre & Marshall, 2012). Second, in seagrass meadows, plants of the same clone stay interconnected by an elaborate rhizome system and share resources at scale estimated of at least some meters (Diaz‐Almela et al., 2007). This can provide an advantage to population dominated by larger clones by reducing the risk of mortality of mats of interconnected plants (Oborny, Kun, Czaran, & Bokros, 2000).

*Agarophyton chilense* is one of the few red algae that has been truly domesticated (Valero et al., 2017), and a strong reduction in genotypic diversity has been observed in Chilean farms (Guillemin et al., 2008). In this species, clones have invaded entire populations and even regions due to vegetative propagation of thallus fragments and exchanges of living material between farms (Guillemin et al., 2008). As all Gracilariaceae, *A. chilense* is characterized by a complex biphasic isomorphic sexual life cycle with both haploid and diploid spores developing a perennial holdfast after settlement on rocky substrate from which grow several fronds. Moreover, it has been shown that *Agarophyton* fronds can live independently and propagate vegetatively when detached from the parental thallus and large asexual populations can develop in muddy or sandy bottom of bays and estuaries (Guillemin et al., 2008; Krueger‐Hadfield et al., 2016). The chosen method of human‐assisted propagation mimics these natural processes and Chilean farmers use successive fragmentation of the thalli to propagate *A. chilense* (Buschmann, Gonzalez, & Varela, 2008). Ultimately, this planting method has potentially selected for the highest growth rates (Guillemin, Sepúlveda, Correa, & Destombe, 2013). Because asexually reproducing thalli can survive and grow for years in soft substrates (Santelices & Doty, 1989), farms of *A. chilense* are dominated by a few clones (Guillemin et al., 2008) and could share important similarities with seagrass meadows of *Zostera marina* and *Posidonia oceanica* (Arnaud‐Haond et al., 2010; Diaz‐Almela et al., 2007).

In the genus *Agarophyton*, controlled experiments in the field where biomass production was measured at distinct genotypic levels in the invasive species *A. vermiculophyllum* (Gerstenmeier et al., 2016) have suggested that complementary effects (due to better resource partitioning and a reduction of competitive overlap among genotypes) could exist in this genus of red algae leading to an enhanced productivity of populations containing more genotypes. However, clonally farmed populations of *A. chilense* have the potential to be dominated by large clones showing high productivity due to the coupling of natural and human‐assisted selection for genotypes that present a high vegetative growth rate. Supporting this idea, selection for general‐purpose genotypes has been suggested in *A. chilense* farms (Usandizaga, Camus, Kappes, Guillemin, & Buschmann, 2019). Monocultures of these general‐purpose genotypes could thus lead to a high biomass production, compensating for the negative effect caused by the low genotypic diversity.

In order to disentangle the possible opposite effects of the loss of genotypic diversity and selection of fast‐growing general‐purpose genotypes in *A. chilense* farms, the aim of our study was to experimentally test for the effect of genotypic diversity on growth in this species. Known genotypes sampled from two different cultivated populations were used in common experimental plots, and growth rate was estimated after one month. In addition to growth measurement, we also assessed the importance of genotypic diversity on other variables that are of importance for cultivation such as epiphyte load, as well as the yield and quality of agar.

## 2 MATERIALS AND METHODS

### 2.1 *Agarophyton chilense* sampling and genotyping

#### 2.1.1 Sampling

**Figure 1:**
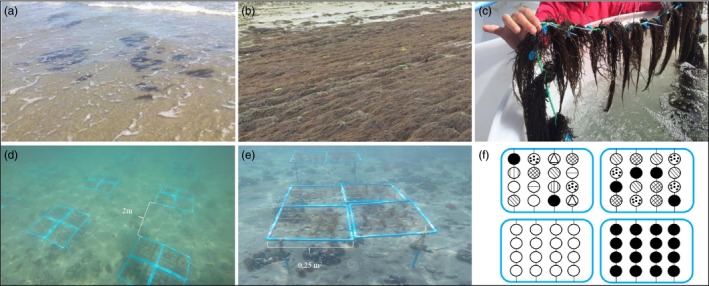
Organization of the field experiment performed at Metri bay. (a) Chaica farm; (b) Ancud farm; (c) Thalli stored in outdoor PVC 1,000 L tanks filled with running filtered seawater; (d) Plots distribution; (e) detail of the four subplots of 0.25 m^2^ each constituting one of our plot; and (f) schematization of the genotypic diversity corresponding to our three treatments: 1 genotype (left and right bottom), 4 genotypes (top right), and 8 genotypes (top left). All photographs by S. Usandizaga and José Luis Kappes

A total of 260 thalli were sampled from two farmed localities: 130 from Ancud, Puente Quilo (41°86′S, 73°98′W) and 130 from Chaica (41°38′S, 72°39′W). Ancud farm is located at the mouth of an estuary, characterized by muddy substrate, while Chaica farm is located in a sandy bay with very strong tidal fluctuations (Figure 1a,b, Figure S1A,C). Cultivation is intensive in the 4 ha of Ancud farm, where thalli are grown on ropes, at high density (Figure 1b). Farming practices in Chaica have been almost abandoned during the last 5 years, and only a small (i.e., more or less one hectare) area, characterized by clumps of *A. chilense* thalli directly buried in sand (Figure 1a), is maintained in this farm. Sampling occurred in September, during the spring season in the southern hemisphere. In order to limit sampling fragments of the same clonal genotype, thalli were separated from each other by at least 10 m.

All thalli were transported from these localities to the Metri Marine Station (CEACIMA) of Universidad de Los Lagos, a site located 30 km southeast of Puerto Montt (41°36′S/72°43′W), Chile. Thalli were then thoroughly cleaned in seawater and stored in 1,000 L outdoor PVC tanks with running filtered seawater (Figure 1c, Figure S1B).

#### 2.1.2 Determination of phase and sex

All fresh thalli were first observed under a Stemi DV4 stereoscopic microscope (Zeiss) to determine phase and sex of mature individuals by direct observation of reproductive structures. Second, for all vegetative thalli, phase and sex were determined using the molecular method reported in Guillemin, Huanel, and Martínez (2012). In brief, both males and females show amplification fragments for only one of the sex markers described. Amplification of the marker named SCAR‐G16‐486 occurs in males while amplification of the marker named SCAR‐D12‐386 occurs only in females (Guillemin et al., 2012). Diploid tetrasporophytes amplified both SCAR‐G16‐486 and SCAR‐D12‐386 bands (Guillemin et al., 2012). All haploid males and females were discarded, and only 119 and 126 diploid tetrasporophytes remained from Ancud and Chaica, respectively. Each tetrasporophyte was then cleaned from all visible epiphytes and marked with a numbered tag sustained by a nylon thread. Marked tetrasporophytes were maintained in the 1,000 L tanks at CEACIMA at 10–12°C and constant aeration during the genotyping (see below).

#### 2.1.3 Genotyping and detection of clones

For each tetrasporophyte, a fragment of approximately 6 cm of cleaned thallus was excised and placed into plastic bags with silica gel for rapid dehydration. Total genomic DNA was extracted following Cohen et al., (2004). Genotyping was made using the six microsatellites described in Guillemin, Destombe, Faugeron, Correa, and Valero (2005), and both PCR reactions mix and amplification program followed the protocols reported by the authors. PCR products were visualized on an ABI 3100 Sequencer fragment analyzer (Applied Biosystem). Allele sizes were scored with GENEIOUS (Biomatters Ltd.), and multilocus genotypes (MLGs) were determined using Genclone v.2.0 (Arnaud‐Haond & Belkhir, 2007).

In Ancud and Chaica, genetic diversity was assessed by computing the percent of polymorphic loci (*P*_99%_), the observed heterozygosity (*H*_o_), the expected heterozygosity (*H*_E_), and alleles per locus (A) with the Genetix software (Belkhir, Borsa, Chikhi, Raufaste, & Bonhomme, 2003). In addition, the *F*_is_ measurement of deviation from random mating was calculated over all loci after running 1,000 permutations using the same software. As suggested by Dorken, Friedman, and Barrett (2002), genotypic diversity was estimated using the Genotypic richness *R*, where *R* = (*G*−1)/(*N*−1), *G* being the number of MLG detected, and *N* the number of genotyped thalli. Genotypic richness was calculated using the software Genclone v.2.0 (Arnaud‐Haond & Belkhir, 2007).

All repeated genotypes were considered as putative fragment of the same clone. For each putative clone, only one sampled thallus was used in the experiment (i.e., the largest one).

Only genotypes showing between 2 and 4 heterozygous loci over the 6 loci studied were used in the experiment (see Table S1). Indeed, it has been suggested that part of the observed effect of genotypic diversity on population growth or resilience could be due to a hidden effect of change in level of genetic diversity (Massa et al., 2013). Since allelic richness of experimental plots rises hand in hand with genotypic diversity, to better control for the possible hidden effect of genetic diversity, genotypes containing very low (<2 heterozygous loci) or very high (>4 heterozygous loci) number of alleles were excluded from our experiment.

### 2.2 Field experiment

To assess whether genotypic diversity influences growth rate, epiphytic load, and agar content, a field experiment was conducted during austral summer (i.e., January and February) 2017 within the sandy bay of Metri located near the CEACIMA. Metri Bay is characterized by fluctuating environmental conditions in terms of water temperature and salinity due to strong tidal currents, and variable winds and freshwater inputs (Buschmann, Kuschel, Vergara, & Schulz, 1992; Buschmann, Westermeier, & Retamales, 1995).

#### 2.2.1 1‐genotype, 4‐genotype and 8‐genotype subplots assembling

Sixteen experimental plots of *A. chilense,* consisting of PVC frames of 1 m^2^, were outplanted. Eight plots were used for each farm. Each plot was divided into four subplots of 0.25 m^2^ within which four nylon ropes were stretched (Figure 1d,e). Following a classical technique used in *A. chilense* cultivation (Halling, Aorca, Cifuentes, Buschmann, & Troell, 2005), within each subplot, 16 thallus fragments were entwined in ropes (four ropes per subplot and four thallus fragments per rope). All thallus fragments were cleaned and weighted at the beginning of the experiment in order to homogenize the size/weight of the fragments to 5 g of fresh algal material. Thalli along the same rope were separated by approximately 2 cm. Subplots supporting three different levels of genotypic diversity were assembled: 1 genotype (16 thallus fragments of only one genotype); 4 genotypes (four different genotypes represented by 4 thallus fragments each); and 8 genotypes (eight different genotypes represented by 2 thallus fragments each) (Figure 1f). Experimental subplots were produced in two steps. First, 16 distinct genotypes from Ancud and 16 distinct genotypes from Chaica were selected at random from the 1,000 L tanks of the CEACIMA to constitute the 1‐genotype subplot (i.e., only thalli weighting more than 110 g were used; 22 × 5 g; 22 thallus fragments = 16 for the 1‐genotype treatment + 4 for the 4‐genotype treatment + 2 for the 8‐genotype treatment, see below). Second, random sampling without replacement was performed to select the *A. chilense* thalli constituting each polyculture‐4 and polyculture‐8 subplot. Each genotype for which a sufficient biomass (>15 g) was left from one round of random sampling was put back in the 1,000 L tank. In both Ancud and Chaica experimental plots, all 16 thalli used in 1‐genotype subplot were also seeded in at least one polyculture‐4 and one polyculture‐8 subplots. Two 1 genotype, one 4‐genotype, and one 8‐genotype subplots were united, using anti‐pullout cable clamps, into experimental plots (see Figure 1c). Distribution of thalli within the subplots and distribution of subplots within the plots were both fully randomized.

#### 2.2.2 Plots installation in the field

Plots were installed in the high subtidal, parallel to the coastline. Plots were fixed on the sandy bottom at a depth of 0.5 m below the average lowest tide. A distance of about 2 m separated each plot from their neighbors (Figure 1d). In order to limit potential micro environmental effects, distribution of plots in the field was changed each week and fully randomized. Moreover, 4 temperature data loggers (HOBO Pendant®, 8K, Onset) recording seawater temperature every 15 min were attached to the plots. Nutrient concentrations (nitrate and phosphate) were analyzed once a week following Strickland and Parson (1972). Plots were kept on the field for 30 days.

#### 2.2.3 Measured variables

Measured variables were chosen to assess the effect of genotypic diversity on (a) parameters that directly influence biomass production and, ultimately, the fitness of the farmed populations of *A. chilense* (i.e., specific growth rate and epiphyte load) and (b) parameters of importance for the commercial value of *A. chilense* in the phycocolloid market (i.e., agar yield and quality).

In the case of the specific growth rate (SGR), the fresh weight of each thalli was measured at the beginning of the experiment and after 30 days in the field with an analytical balance (accuracy 0.0001 mg, Radwag) and it was calculated as the percentage of wet weight gain per day according to the formula: SGR = [ln (*W*_f_/*W*_i_)/(*t*_f_‐*t*_i_)] × 100; where SGR = specific growth rate, *W*_i_ = initial fresh weight, *W*_f_ = final fresh weight, and *t* = time (days).

After 30 days, the experiment was ended and all samples were transported, slightly wet, to the laboratory within an isothermal cooler crate. Once at the laboratory, 3 thalli were randomly selected per subplot, and for each of them, all visible epiphytes were removed. A simple taxonomic classification was intended for the epiphytes while examining their structural characteristics following the descriptions provided by Hoffmann and Santelices (1997). Epiphytes were classified under four taxonomic groups: *Ulva* spp., *Polysiphonia* spp., *Ectocarpus* spp., and *Rhizoclonium* spp. For each epiphyte group found, its wet weight was measured after a slight blotting and using a digital balance (accuracy 0.0001 mg, Radwag). The epiphytic load was determined as the weight (g) of epiphytes/weight (g) *A. chilense* (measures are given for *Ulva* spp.*, Ectocarpus* spp. and total epiphyte load). Finally, all fresh material remaining from each subplot was joined and agar yield and quality (i.e., gel strength) and was measured following the method of Cancino, Muñoz, and Orellana (1987).

### 2.3 Statistical analysis

Effects of genotypic diversity (1 genotype, 4 genotypes and 8 genotypes) and locality of origin (i.e., Ancud and Chaica) on five of the six measured variables (*Ulva* spp. epiphyte load, *Ectocarpus* spp. epiphyte load, total epiphyte load, gel strength, and agar yield) were tested by a two‐way ANOVA followed by the Tukey multiple comparison tests. Effects of genotypic diversity (G.T) and locality of origin (L.O) were fixed and fully crossed (G.T × L.O). The “subplot” was used as experimental unit in our analyses. For each locality of origin of the thalli sampled, eight replicates of the experimental unit were available for 4‐genotype and 8‐genotype treatments and 16 for 1‐genotype treatment. When nonnormal residuals and heterocedasticity were detected, data were logarithmically transformed (for: *Ulva* spp. and *Ectocarpus* spp. epiphyte load, and total epiphyte load) prior to analyses. To test for the effects of genotypic diversity and locality of origin on the mean specific growth rate (SGR), two‐way ANOVA permuted 1,000 times (or PERANOVA, Anderson, 2001) was used since nonnormal residuals and heterocedasticity remained even after data transformation for this variable.

The effect of genotypic diversity on growth rate was evaluated for each genotype used (i.e., 16 genotypes from Ancud and 16 genotypes from Chaica, see Table S2) in both monoclonal and polyclonal experiment plots using one‐way ANOVA, followed by the Tukey comparison tests. We only retained 1‐genotype and 4‐genotype treatments in order to keep at least 4 repetitions per treatment to test the factor genotypic diversity. Genotypic diversity was considered as a fixed factor for each of the localities of origin, which were analyzed independently.

All analyses were performed in R (3.2.4 version) (R Core Team, 2016).

## 3 RESULTS

### 3.1 Characteristics of the two sampled farms

Both farmed populations were composed mainly of diploid individuals: 79% of tetrasporophytes, 12% of males and 9% of females in Ancud, 97% of tetrasporophytes, 2% of males and 1% of females in Chaica. A total of 20 alleles were detected in Ancud and 16 in Chaica, with 15 alleles shared between localities. Allelic diversity was higher in Ancud than in Chaica (values for average number of alleles per locus (A) were 3.33 and 2.67, respectively, Table 1) but these differences were nonsignificant (Mann–Whitney test calculated using unilocus values of A and He; *p*‐value > .18). From the 228 *A. chilense* tetrasporophyte thalli collected and genotyped for all six microsatellite loci, a total of 71 different genotypes were detected in Ancud and 43 in Chaica. Genotypic diversity (*R*) varied noticeably among the populations, with the lowest value observed in Chaica (*R* = .33 lower than 0.55 in Ancud; Table 1). Mean expected heterozygosity was higher than 0.4 in both farmed populations (Table 1). Positive and significant *F*_is_ values were detected in both farms (Table 1).

**Table 1:** Genetic diversity estimated in the two sampling farms

Farmed populations	*N*	♀	♂	Tetra	*P*_99%_	*A*	*H*_E_	*H*_O_	*F*_is_	*R*
Ancud	130	12	15	103	100	3.333	0.425	0.314	0.266***	0.546
Chaica	130	1	3	126	100	2.667	0.541	0.432	0.206***	0.331

Note

Percent of polymorphic loci (*P*_99%_ criterion), average number of alleles per locus (A), expected heterozygosity (*H*_E_), observed heterozygosity (*H*_o_), and departure from Hardy–Weinberg equilibrium (*F*_is,_ *p*‐value given for 1,000 permutation, ***p < .001) and the genotypic richness (*R*, with *R* = number of multilocus genotypes detected/ number of genotyped thalli) were calculated within each population. *N* = total number of samples. ♀ = females (*n*) and ♂ = males (*n*), haploid individuals were not genotyped. Tetra = number of genotyped tetrasporophytes (2n) for which all 6 microsatellite loci were amplified.

### 3.2 Environmental parameters

During the experiment, seawater temperature was high for South Chile with an average value of 19.2°C ± 1.0. Nitrate concentrations were always lower than 4.00 µM, and phosphate concentrations were relatively constant with an average value of 0.06 µM ± 0.00.

### 3.3 Growth rate

**Figure 2:**
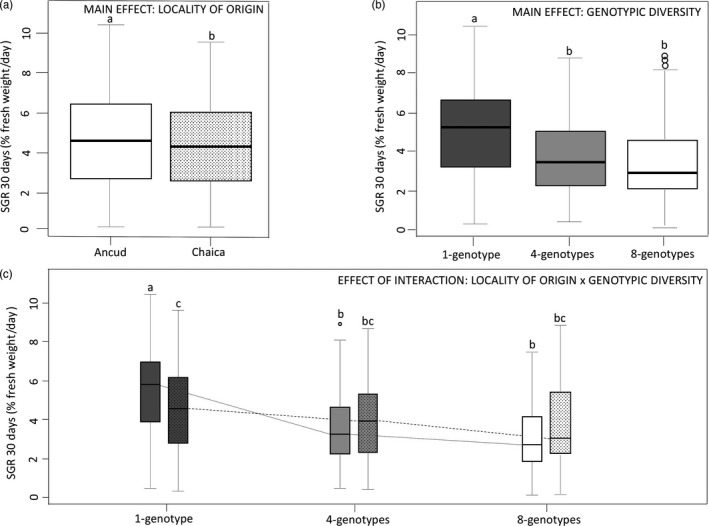
Main effects of population type and genotypic diversity and the interaction between these two factors on the specific growth rate (SGR) of *Agarophyton chilense*. (a) Main effect of the locality of origin (Ancud and Chaica; L.O); (b) Main effect of the genotypic diversity treatments (Monoclonal: 1 genotype and polyclonal: 4 genotypes and 8 genotypes; G.T.); and (c) Effect of the interaction between the locality of origin and genotypic diversity (L.O × G.T). Lowercase letters indicate differences at *p* < .05 for the Tukey multiple comparison tests. Grayscale represent the genotypic diversity (dark‐gray: monoclonal treatment; gray and white: polyclonal treatments (4 genotypes and 8 genotypes, respectively) and fill pattern the locality of origin (no pattern: Ancud; dots: Chaica). Box plots on the left and right of the figure depict the overall differences between genotypic treatments. Box plot whiskers show the 1%–99% range values; the horizontal line in each box plot shows the median, and the colored segment shows the quartile range (25%–75%). Values outside of the whisker range are plotted as dots

**Figure 3:**
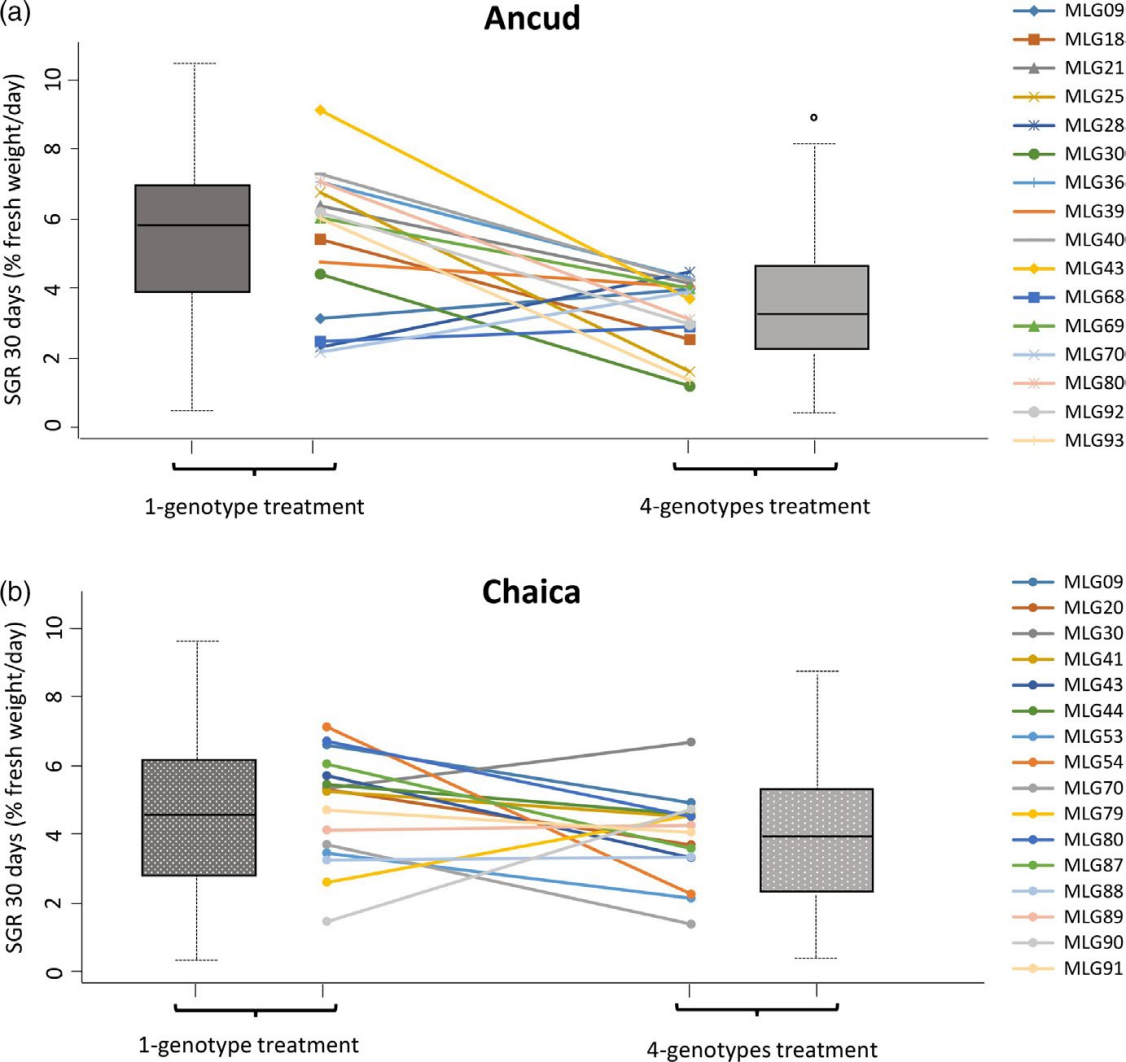
Change in specific growth rate (measured as % wet weight/day) of each 32 *Agarophyton chilense* genotypes (A: 16 genotypes from the locality of Ancud; no fill pattern; B: 16 genotypes from the locality of Chaica; fill pattern: dots) grown in two distinct genotypic diversity treatments: 1 genotype and 4 genotypes (see Section 2). Box plots on the left and right of the figure depict the overall differences between genotypic treatments. Box plot whiskers show the 1%–99% range values; the horizontal line in each box plot shows the median, and the colored segment shows the quartile range (25%–75%). Values outside of the whisker range are plotted as dots. The reaction norm, for each genotype, is presented between box plots; color represents genotype identity

Significant main effects of locality of origin (*F*_1,834_ = 5.65; *p* = .02) and genotypic treatment (*F*_2,833_ = 44.08; *p* = .001) were detected on the specific growth rate (SGR). Interaction between these two factors was also significant (*F*_2,826_ = 15.48; *p* = .001) (Table 2, Figure 2). In Ancud, SGR was significantly higher for the monoclonal than for the polyclonal treatments (Figure 2c). The same general pattern was observed in Chaica (Figure 2c); with *p*‐values estimated for HSD (honest significant difference) tests being close to significance (*p*‐value = .101 and .088, calculated between 1‐genotype and 4‐genotype and between 1‐genotype and 8‐genotype treatments, respectively). Supporting these results, the reaction norm observed for *A. chilense* genotypes grown in distinct genotypic diversity treatments (Figure 3) show a clear tendency of SGR decrease between 1‐genotype and 4‐genotype treatments in Ancud (SGR decrease significant for 11 of the 16 genotypes studied; Table S2) while the pattern was less clear in Chaica (significant decrease in SGR between 1‐genotype and 4‐genotype treatments for only 4 of the 16 genotypes studied; Table S2).

**Table 2:** Two‐way analysis of variance (ANOVA) on the *Agarophyton chilense* specific growth rate (SGR) (A), total epiphytic load (B), *Ectocarpus* spp. load (C), *Ulva* spp. load (D), quality of agar measured as gel strength (E), and agar yield (F)

Source	*df*	SS	*F*	*P*
(A) Specific growth rate				
Genotypic treatment (G.T)	2	385.5643	54.1649	.001**
Locality of origin (L.O)	1	25.7130	7.2244	.043*
G.T × L.O	2	110.2070	15.4821	.001**
Error	826	3,617		
(B) Total epiphytic load				
Genotypic treatment (G.T)	2	0.001	0.0002	.9997
Locality of origin (L.O)	1	16.372	7.1412	.00870**
G.T × L.O	2	7.373	1.6081	.20509
Error	108	247.596		
(C) *Ectocarpus* spp. load				
Genotypic treatment (G.T)	2	1.245	0.3888	.68032
Locality of origin (L.O)	1	9.159	5.7218	.02131*
G.T × L.O	2	7.284	2.2754	.11528
Error	42	67.229		
(D) *Ulva* spp. load				
Genotypic treatment (G.T)	2	1.386	0.3355	.718914
Locality of origin (L.O)	1	26.137	12.6553	.001974**
G.T × L.O	2	3.113	0.7535	.483601
Error	20	41.306		
(E) Gel strength				
Genotypic treatment (G.T)	2	<0.0001	1.378	.328
Locality of origin (L.O)	1	<0.0001	0.045	.833
G.T × L.O	2	<0.0001	1.182	.314
Error	58	<0.0001		
(F) Agar yield				
Genotypic treatment (G.T)	2	31.29	0.6799	.5112
Locality of origin (L.O)	1	49.88	2.1678	.1471
G.T × L.O	2	37.00	0.8039	.4532
Error	51	1,173.56		

Note

*Agarophyton chilense* thalli were sampled from two localities (L.O; Ancud and Chaica), and three distinct genotypic diversity treatments were applied (G.T; 1 genotype, 4 genotypes and 8 genotypes). The two factors are fixed and fully crossed. *p < .05; **p < .01; ***p < .001.

### 3.4 Epiphyte load

A significant effect of the locality of origin was detected (*F*_1,108_ = 7.14; *p* = .009 for the whole epiphytic load, Table 2). Overall, the epiphytic load was low (total load <0.30 g epiphyte/g *A. chilense*, Table 3). Four different genera of epiphytes appeared on *A. chilense* thalli during the experiment: *Ectocarpus* spp.*, Ulva* spp.*, Polysiphonia* spp*.,* and *Rhizoclonium* spp. *Ulva* spp. epiphytes were ten times more abundant in Chaica, with a load up to 0.059 g epiphyte/g *A. chilense,* than in Ancud (Table 3). *Ectocarpus* spp. load was slightly higher in Ancud (with an average of 0.045 g epiphyte/g *A. chilense*) than in Chaica (with an average of 0.028 g epiphyte/g *A. chilense*) (Table 3). Similarly, total epiphyte load was generally higher in Ancud (with an average of 0.133 g epiphyte/g *A. chilense*) than in Chaica (with an average of 0.088 g epiphyte/g *A. chilense*, Table 3). Unexpectedly, no significant effect of genotypic diversity was detected on epiphytic load (*F*_2,108_ = 0.00; *p* = .100; Table 2).

**Table 3:** Yield and quality of agar and epiphytic load measured in *Agarophyton chilense* thalli from Ancud and Chaica and summited to three distinct genotypic diversity treatments (i.e., monoclonal: 1 genotype and two polyclonal: 4 genotypes and 8 genotypes). (A) Specific growth rate (SGR) measured as % wet weight/day, (B) Epiphytic load measured as g epiphytes/g *A. chilense* (measures are given for *Ulva* spp.*, Ectocarpus* spp. and total epiphyte load), and (C) quality of agar (gel strength, g/cm^2^) and agar yield (%)

Response variables	ANCUD			CHAICA		
	Monoclonal	Polyclonal		Monoclonal	Polyclonal	
	1 genotype	4 genotypes	8 genotypes	1 genotype	4 genotypes	8 genotypes
(A)						
SGR	5.496 ± 2.263^a^	3.485 ± 1.876^b^	3.020 ± 1.591^b^	4.568 ± 2.124^c^	3.928 ± 1.982^bc^	3.892 ± 2.173^bc^
(B)						
Total epiphyte load	0.112 ± 0.001^a^	0.153 ± 0.002^ab^	0.134 ± 0.001^ab^	0.078 ± 0.001^b^	0.112 ± 0.002^ab^	0.075 ± 0.001^ab^
*Ulva* spp. load	0.002 ± 0.000^a^	0.004 ± 0.000^ab^	0.003 ± 0.000^ab^	0.046 ± 0.001^b^	0.059 ± 0.001^ab^	0.057 ± 0.001^ab^
*Ectocarpus* spp. load	0.053 ± 0.001^a^	0.035 ± 0.001^a^	0.048 ± 0.001^a^	0.020 ± 0.000^a^	0.056 ± 0.001^a^	0.008 ± 0.000^a^
(C)						
Gel strength	698.07 ± 165.97^a^	828.62 ± 212.29^a^	683.52 ± 203.53^a^	771.10 ± 234.78^a^	701.76 ± 340.16^a^	615.97 ± 227.34^a^
Agar yield	16.41 ± 4.87^a^	13.19 ± 4.28^a^	16.00 ± 6.04^a^	13.47 ± 4.43^a^	13.63 ± 3.30^a^	15.64 ± 5.83^a^

Note

Data are means ± *SD*. For each locality of origin of the thalli sampled, eight replicates of the experimental unit were available for the polyclonal treatments and 16 for the monoclonal. Distinct uppercase letters denote significant differences after the Tukey test.

### 3.5 Yield and gel strength of agar

Agar quality (measured as gel strength) and yield were not significantly affected by genotypic diversity nor by locality of origin (Table 2). Very similar values of yield and gel strength were detected among localities (yield: from 13.19% to 16.41% in Ancud and from 13.47% to 15.64% in Chaica; gel strength: from 683.52 ± 203.53 g/cm^2^ to 828.62 ± 212.29 g/cm^2^ in Ancud and from 615.97 ± 227.34 g/cm^2^ to 771.10 ± 234.78 g/cm^2^ in Chaica; Table 3).

## 4 DISCUSSION

Results obtained in *A. chilense* tend to conflict part of the literature reporting experimental support for a positive effect of genotypic diversity on the resilience and resistance of populations. The present work shows better performances of monoclonal stands over polyclonal ones for growth rate after one month in the field. This suggests an underlying effect of farming practices leading on the one hand to decreased genotypic diversity and on the other hand to selection of fast‐growing competitive large clones in *A. chilense* Chilean farms. Significant effect of the locality of origin detected on epiphytic load and growth rate of thalli point out to possible ecological differences between *A. chilense* from Ancud and Chaica; these may be linked to fishermen's cultivation techniques or environment variables characteristic of each farm. Agar production was not affected by origin of the thalli nor by genotypic diversity of the plots. Finally, knowing the effect of enhanced epiphyte load, results obtained here may inspire longer term farming strategies to optimize the potential trade‐off between short‐ and long‐term effects of genotypic diversity in response to environmental variations and pest infections.

### 4.1 Selection for fast‐growing competitive large clones in A. chilense farms and their unexpected negative effect on genotypic diversity

Unexpectedly, the results demonstrate that the level of genotypic diversity can affect *A. chilense* specific growth rate, yet the opposite is usually reported in the literature. Monoclonal subplots showed a significantly higher growth rate than multiclonal ones in *A. chilense*, this trend contrasting with the better growth, resistance or resilience reported for most plant species (*Zostera marina*: Ehlers et al., 2008; Hughes & Stachowicz, 2004; Reusch et al., 2005; *Arabidopsis thaliana*: Kotowska, Cahill, & Keddie, 2010) as well as the results on productivity for the only species of red alga submitted to this kind of manipulative experiment so far, *A. vermiculophyllum* (Gerstenmaier et al., 2016). However, in this last species, the positive effects of genotypic diversity were only detected in the mid intertidal during summer, when individuals were submitted to the most stressful environmental conditions while no effect was observed when the patches were planted during winter, or in the low intertidal whatever the season. The authors proposed that the positive effects of genotypic diversity only arise when *A. vermiculophyllum* was grown in stressful environments (Gerstenmaier et al., 2016). One could argue that the environmental settings in Metri were extremely favorable to *A. chilense* growth and that the temperature and nutrient concentration during the time of the experiment has limited our capacity to detect positive effects of genotypic diversity. Nevertheless, this explanation does not account for the significantly higher growth rate of the 1‐genotype subplot compared with 4‐genotype subplots. Moreover, an experiment conducted during the same period in the Metri Bay detected nutrient limitation for growth in *A. chilense*, supporting the fact that the environmental conditions were not actually ideal for the species during our study (Usandizaga, Camus, Kappes, Guillemin, & Buschmann, 2018).

In contrast to the other studies mentioned above, *A. chilense* has a history of domestication (see Buschmann et al., 1995; Santelices & Doty, 1989) in which the fishermen's farming activities could have favored genets presenting a high vegetative growth rate and high biomass production when grown in monoclonal culture. Other studies, in trees used for wood production, concluded also that identical or even higher yields could be obtained by planting large monoclonal stands rather than plots composed by mosaics of distinct genotypes (Coyle, McMillin, Hall, & Hart, 2002; DeBell & Harrington, 1997). These results have been linked to the potential competition between neighboring unrelated genotypes and the difficulty to have access to the dozens of not related, high‐quality clones, equality suited to the soil type necessary to obtain a biomass production in polyclonal plots at least identical to the one obtained in monoclonal plots of the most productive clones (Coyle et al., 2002). We propose that selection of genotypes showing high biomass productivity in a competitive environment could be ongoing in farms, especially the ones where more intensive cultivations methods are applied. It is also possible that natural selection in particular sites (e.g., the ones presenting high density) could lead to the spread of multipurpose genotypes showing high SGR even when growing in mostly or even purely clonal stands. Fast‐growing competitive large clones could slowly invade farms leading to populations ultimately more resistant to perturbations than more diverse populations consisting of many little clones, counterbalancing then the reported positive effect of the genotypic diversity (Diaz‐Almela et al., 2007; Reusch & Hughes, 2006). Similar results have also been obtained in natural populations of partially asexual organisms and related to the selection of generalist clones able to cope with highly variable environment. For example, in rotifers, no positive effect of genotypic diversity, through diversification in resource use, on population growth was detected (Dimas‐Flores, Serra, & Carmona, 2013). In *Posidonia oceanica*, lower mortality rates have been reported in meadows with lower genotypic and genetic diversity subjected to aquaculture impacts (Diaz‐Almela et al., 2007). Large *P. oceanica* genotypes seem to resist better to fish farm‐derived impacts than little ones. Possible explanations that could account for this effect include clonal integration (resources sharing), foraging advantage (capacity to explore a larger range of different micro‐habitats by the same genetic individual when its number of modular unit increases, optimizing its capacity to reach micro‐environments where it is better adapted) or other size‐related fitness traits (e.g., dominance of the fittest genotypes). The authors suggested that the effects of clonal size structure could play an important role on meadow survival. Resource sharing between *A. chilense* clones is not possible since the genets propagating vegetatively are never physically connected by stolon, or rhizomes at large scale as in other clonal organisms.

Another possible explanation to the discrepancy between our results and the classically positive effect of genotypic diversity reported on population growth or resilience is the fact that our experimental design explicitly limited the range of genetic diversity present in the different plots, in order to limit confounding effects of genotypic and genetic diversity. Most of the studies reported above did not disentangle the impact of the levels of genotypic and genetic diversity. In fact, the one that did (Massa et al., 2013) showed they are very often highly correlated in natural populations, resulting in a possible hidden effect of genetic diversity in some of the previously reported experiments. Indeed, the only significant effect of genotypic richness in this study was negative (Massa et al., 2013).

### 4.2 Differences in ecological settings and fishermen's management strategy between farms and their putative impacts on genotypes selection

Even if we propose that potential selection of general‐purpose genotypes, presenting high growth rate in agroecosystems controlled by farmer's activities, exists in most/all Chilean *Agarophyton* farms, the two farms under study clearly presented important differences. Indeed, we detected a significant effect of the locality of origin on epiphytic load and growth rate. Differences in fishermen's cultivation techniques or environmental variables could have led to ecological differences between *A. chilense* from Ancud and Chaica. Even if only separated by 130 km, the two localities are established in quite distinct habitats. Ancud is located at the mouth of an estuary characterized by muddy substrate, while Chaica is located in a sandy bay with very strong tidal influence. Moreover, in Ancud, cultivation is intense and based at least in part on the use of ropes inoculated by diploid spores added to the farmed thalli. In this case, each year, the fishermen collect fertilized haploid female from nearby natural populations and use them to seed ropes (Alveal, Romo, Welinger, & Oliveira, 1997). This technique allows first, to integrate new genotypes generated by sexual reproduction and second, to grow *A. chilense* thalli in very high density along the ropes. For example, the spore‐seeding method developed by Alveal et al., (1997) showed a high production potential with 6.5 kg/m after 15 months of cultivation under protected estuarine conditions. The farm of Chaica, on the other hand, is small, use extensive farming techniques and is characterized by fairly spaced clumps of *A. chilense* thalli buried in sand (authors pers. obs.). Besides, it is seeded mostly by the incorporation of floating thalli coming from the same bay, or directly from the own farm, and produced by vegetative fragmentation. This fact could explain the reduced number of alleles and genotypic richness founded in this farm when compared to Ancud. These differences in ecological settings and fishermen's management strategy could have led to distinct selective processes in the two farms. However, at first sight, we could have expected a stronger selection for long‐lived multipurpose genotypes in Chaica due to the more stable setups linked to the farming practice. Nonetheless, it has to be noted that the rate of genotypes turnover/ rate of incorporation of new genotypes by seedlings (in Ancud) or by drifting thalli (in both Ancud and Chaica) have not been studied yet in these two farms.

### 4.3 Ancud and Chaica, two farms with distinct susceptibility to epiphytes but similar agar yield and quality

According to the literature, clonal crops are also likely to be less able to cope with the outbreak of new diseases (Provan et al., 1999). Reduced genotypic diversity is known to affect the ability of populations to cope with parasitic and infectious diseases. Indeed, parasite pressure and the Red Queen hypothesis have been advanced as one of the main mechanism contributing to the persistence of sexual reproduction in partially asexual organisms, allowing the rapid integration of new genotypes in a population of parasites adapted to the most common hosts (Altermatt & Ebert, 2008; King et al., 2011). Unexpectedly, effect of genotypic diversity on epiphytic load was not detected in our study. Likewise, in *A. vermiculophyllum*, no effects of genotypic diversity on epiphyte, bacterial or epifaunal abundance, or on invertebrate diversity were reported (Gerstenmaier et al., 2016). Bishop and Byers, (2015) and Kollars, Byers, and Sotka (2016) stated that the organisms inhabiting the algal mats do not impact the fitness of their host since they use it as habitat for protection from consumers or abiotic stress. However, another explanation could be that the duration of the experiment was too short to detect a significant effect of genetic diversity on epiphytic load (but see contrasting results obtained in short‐term experiments in Metri Bay by Kuschel & Buschman, 1991). Indeed, the positive effect of genetic diversity to reduce the spread of host populations is sometime not detected at the beginning of the experiments but only after a substantial period of growth (Lively, 2010; Schmid, 1994). This could be linked to disease/pest buildup over time and, indeed, crop rotation is a common pest management tactic used in agriculture, in particular in woody trees mostly planted in monoclonal blocks or rows (Coyle et al., 2002).

However, we did detect differences in terms of load and epiphyte genera associated with the *Agarophyton* thalli depending on the locality of origin. Results showed that epiphyte load was higher in Ancud than in Chaica and that thalli from Chaica were more epiphyted by *Ulva* spp. whereas *Ectocarpus* spp. were the epiphytes mostly observed on Ancud thalli. Differences in incidence and prevalence of epiphyte community and/or in resistance among thalli collected in distinct regions have also been observed in *A. vermicullophyllum* (Wang et al., 2016). Evidence suggests that seaweeds from distinct origins, but cultivated under the same conditions in the field can show differences in morphology or chemical constituents (Buschmann et al., 1992; Hanisak, Littler, & Littler, 1990). Chemical defenses against algal macrofoulers have also been reported for *A. chilense,* suggesting that relevant concentrations of oxylipins reduced spore settlement of *Acrochaetium* sp. (Rhodophyta, Acrochaetiaceae) and suppressed the development of hapteria in *Ceramium rubrum* (Rhodophyta, Ceramiaceae). In *A. vermiculophyllum* extractable surface‐bound metabolites have been shown to mediate the defenses of the species against *Ceramium* filaments (Wang et al., 2016). We propose that local differences in chemical defense mechanisms between *A. chilense* thalli could have contributed to the difference between the two farms concerning epiphyte load. The presence of thalli with distinct susceptibility to epiphyte infection has already been reported in *A. chilense.* Indeed, the study of Buschmann et al., (1992) show that different populations of *Agarophyton* grown under the same experiment conditions maintain different susceptibility to epiphytism even after several month of cultivation. Similar results were obtained by Santelices and Ugarte, (1990) who reported different responses to epiphytes (sensitivity to epiphytes) among *Agarophyton* populations. Finally, because of the significance of the agar yield and quality for the commercial value of *A. chilense* in the phycocolloid market, it is important to highlight that little information exists on the effect of genetic factors on the thalli agar production. The agar yield and gel strength of *A. chilense* can differ significantly depending on the locality of origin (Buschmann et al., 1992). However, no significant effect of genotypic diversity or locality of origin on agar production and gel quality was detected in the present experiment. Experiments following the agar production and gel quality of particular genets should be considered in future research.

## 5 CONCLUSION

The drastic genotypic variability reduction reported in *Agarophyton chilense* farmed populations, due essentially to farmers clonal propagation practices, have been proposed to be in part linked to the recent decrease in farms productivity (Usandizaga et al., 2018). Even if no significant effect of genotypic diversity on epiphyte load was observed in *A. chilense*, it is still important to take into account that in monoclonal plots; once a disease/pest becomes established, it can proliferate very rapidly throughout the entire plot leading to enormous economic loss (Coyle et al., 2002; Kuschel & Buschmann, 1991). However, various genotypes, especially the ones growing in Ancud, grow significantly better in monoclonal than in polyclonal plots. In order to maintain a high growth rate in *A. chilense* without jeopardizing the future of the crops, we suggest the implementation of a classical design of clonally propagated tree plantations, where farms should be mosaics of monoclonal stands within which selected high‐quality clones are planted and a rotation of the genotypes is implemented through space and time. This last management measure may become an efficient strategy to ensure a sufficient diversity to resists to longer term environmental fluctuations and limit pest buildup in the fields over time.

## CONFLICT OF INTEREST

No potential conflict of interest was reported by the authors.

## Data Availability

All data supporting this study are provided in the results section and as supplementary information accompanying this paper.
