# Supplementary figures and images for: Better off alone? Compared performance of monoclonal and polyclonal stands of a cultivated red alga growth
Source: Evol Appl. 2020 Jan 22;13(5):905–17. doi: 10.1111/eva.12908 (PMC7232761; doi:10.1111/eva.12908)

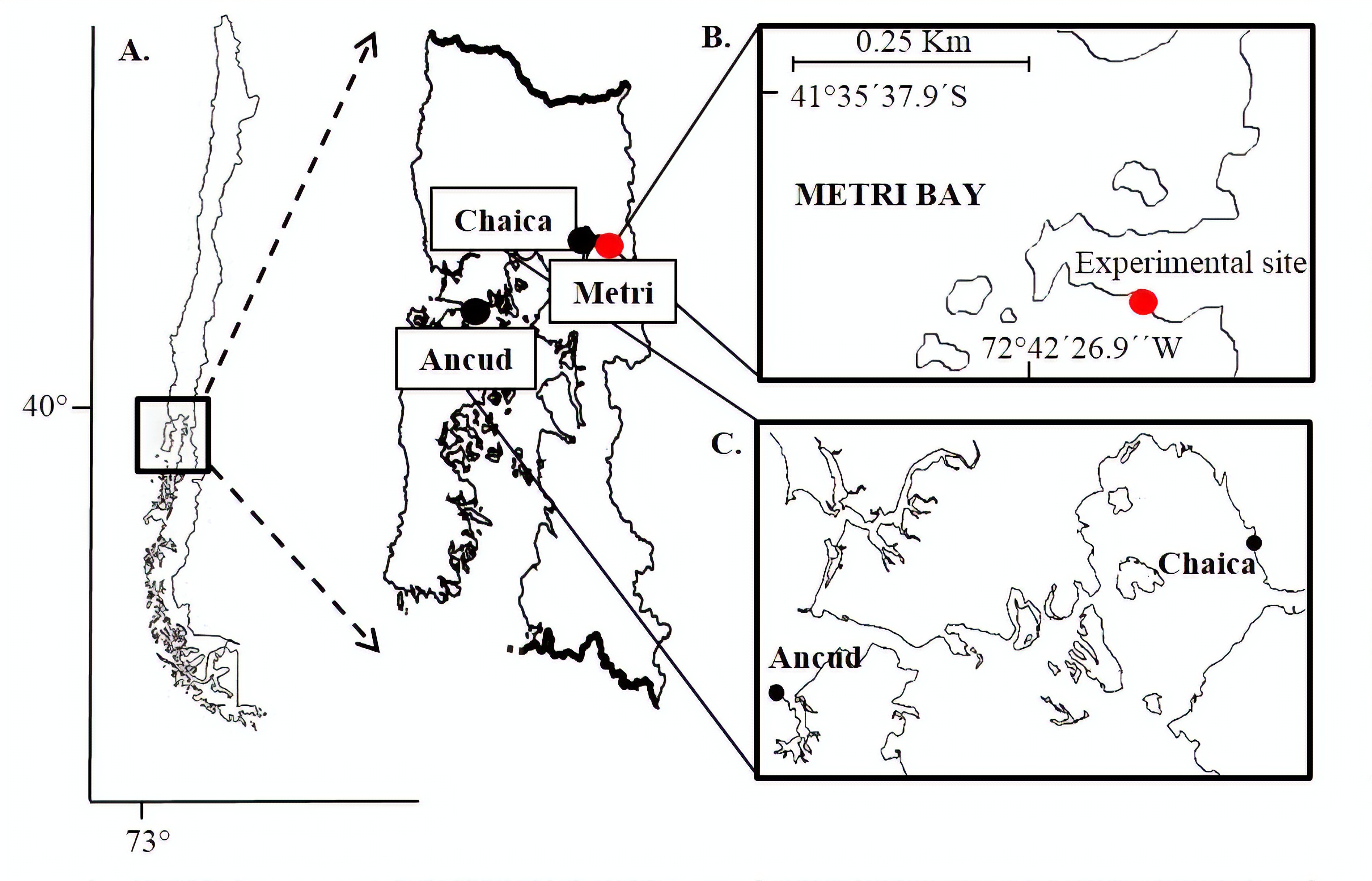

Supplement: Supplementary file 1 [file EVA-13-905-s001.jpg]
