# Supplementary material for: Better off alone? Compared performance of monoclonal and polyclonal stands of a cultivated red alga growth
Source: Evol Appl. 2020 Jan 22;13(5):905–17. doi: 10.1111/eva.12908 (PMC7232761; doi:10.1111/eva.12908)
Supplement: Supplementary file 2 [file EVA-13-905-s002.docx]

**Supplementary Table 1:** Genotype profile of 229 tetrasporophytes (i.e., diploids) of *Agarophyton chilense* collected at two sites (the Ancud and Chaica farms) and scored for six microsatellite markers. MultiLocus Genotypes (MLGs) were determined using Genclone v.2.0 (Arnaud-Haond & Belkhir, 2007). * indicate the genotypes used in the experiment.

| **Sampling site** | **Sample code** | **MLGs** | **Locus Grc-2B2** | | **Locus Grc-6C7** | | **Locus Grc-7D3** | | **Locus Grc-7F12** | | **Locus Grc-**  **8B2** | | **Locus Grc-AC/CT23** | |
| --- | --- | --- | --- | --- | --- | --- | --- | --- | --- | --- | --- | --- | --- | --- |
| Ancud | ANCUD_01 | MLG25* | 302 | 316 | 197 | 197 | 261 | 297 | 201 | 203 | 357 | 357 | 222 | 222 |
| Ancud | ANCUD_02 | MLG23 | 302 | 316 | 197 | 197 | 261 | 261 | 203 | 203 | 357 | 357 | 222 | 222 |
| Ancud | ANCUD_03 | MLG65 | 316 | 316 | 197 | 197 | 261 | 261 | 203 | 203 | 357 | 357 | 220 | 220 |
| Ancud | ANCUD_04 | MLG14 | 302 | 302 | 197 | 197 | 300 | 300 | 201 | 201 | 357 | 357 | 222 | 222 |
| Ancud | ANCUD_05 | MLG85 | 318 | 318 | 197 | 197 | 261 | 261 | 201 | 201 | 361 | 361 | 222 | 222 |
| Ancud | ANCUD_06 | MLG28* | 302 | 316 | 197 | 197 | 261 | 300 | 201 | 201 | 357 | 357 | 222 | 222 |
| Ancud | ANCUD_07 | MLG14 | 302 | 302 | 197 | 197 | 300 | 300 | 201 | 201 | 357 | 357 | 222 | 222 |
| Ancud | ANCUD_09 | MLG92* | 318 | 318 | 197 | 201 | 261 | 261 | 192 | 201 | 357 | 361 | 222 | 222 |
| Ancud | ANCUD_10 | MLG23 | 302 | 316 | 197 | 197 | 261 | 261 | 203 | 203 | 357 | 357 | 222 | 222 |
| Ancud | ANCUD_11 | MLG29* | 302 | 316 | 197 | 197 | 261 | 300 | 201 | 203 | 357 | 357 | 220 | 220 |
| Ancud | ANCUD_12 | MLG03 | 302 | 302 | 197 | 197 | 261 | 261 | 201 | 201 | 357 | 357 | 222 | 222 |
| Ancud | ANCUD_13 | MLG69* | 316 | 316 | 197 | 197 | 261 | 300 | 192 | 203 | 357 | 361 | 222 | 222 |
| Ancud | ANCUD_16 | MLG59 | 314 | 314 | 197 | 197 | 300 | 300 | 201 | 201 | 357 | 361 | 222 | 222 |
| Ancud | ANCUD_18 | MLG70 | 316 | 316 | 197 | 197 | 261 | 300 | 201 | 203 | 357 | 357 | 222 | 222 |
| Ancud | ANCUD_19 | MLG70 | 316 | 316 | 197 | 197 | 261 | 300 | 201 | 203 | 357 | 357 | 222 | 222 |
| Ancud | ANCUD_21 | MLG93 | 318 | 318 | 197 | 201 | 297 | 300 | 201 | 203 | 361 | 361 | 222 | 222 |
| Ancud | ANCUD_22 | MLG93* | 318 | 318 | 197 | 201 | 297 | 300 | 201 | 203 | 361 | 361 | 222 | 222 |
| Ancud | ANCUD_23 | MLG22* | 302 | 316 | 197 | 197 | 261 | 261 | 201 | 203 | 357 | 357 | 222 | 222 |
| Ancud | ANCUD_25 | MLG21* | 302 | 316 | 197 | 197 | 261 | 261 | 192 | 203 | 357 | 361 | 222 | 222 |
| Ancud | ANCUD_26 | MLG93 | 318 | 318 | 197 | 201 | 297 | 300 | 201 | 203 | 361 | 361 | 222 | 222 |
| Ancud | ANCUD_27 | MLG10 | 302 | 302 | 197 | 197 | 261 | 300 | 203 | 203 | 357 | 357 | 222 | 222 |
| Ancud | ANCUD_28 | MLG09 | 302 | 302 | 197 | 197 | 261 | 300 | 201 | 203 | 357 | 357 | 222 | 222 |
| Ancud | ANCUD_29 | MLG03 | 302 | 302 | 197 | 197 | 261 | 261 | 201 | 201 | 357 | 357 | 222 | 222 |
| Ancud | ANCUD_30 | MLG52* | 302 | 318 | 197 | 201 | 261 | 300 | 201 | 201 | 357 | 357 | 222 | 222 |
| Ancud | ANCUD_31 | MLG66 | 316 | 316 | 197 | 197 | 261 | 261 | 203 | 203 | 357 | 357 | 222 | 222 |
| Ancud | ANCUD_33 | MLG81 | 316 | 318 | 197 | 201 | 261 | 297 | 192 | 201 | 357 | 361 | 222 | 222 |
| Ancud | ANCUD_36 | MLG11 | 302 | 302 | 197 | 197 | 297 | 300 | 201 | 201 | 357 | 357 | 220 | 220 |
| Ancud | ANCUD_38 | MLG67* | 316 | 316 | 197 | 197 | 261 | 263 | 201 | 205 | 357 | 357 | 220 | 220 |
| Ancud | ANCUD_39 | MLG78* | 316 | 318 | 197 | 197 | 261 | 300 | 201 | 203 | 357 | 361 | 222 | 222 |
| Ancud | ANCUD_41 | MLG36* | 302 | 316 | 197 | 197 | 300 | 300 | 201 | 203 | 357 | 357 | 220 | 220 |
| Ancud | ANCUD_42 | MLG30 | 302 | 316 | 197 | 197 | 261 | 300 | 201 | 203 | 357 | 357 | 222 | 222 |
| Ancud | ANCUD_43 | MLG68* | 316 | 316 | 197 | 197 | 261 | 300 | 192 | 201 | 357 | 357 | 222 | 222 |
| Ancud | ANCUD_44 | MLG70* | 316 | 316 | 197 | 197 | 261 | 300 | 201 | 203 | 357 | 357 | 222 | 222 |
| Ancud | ANCUD_45 | MLG56 | 302 | 318 | 197 | 201 | 297 | 300 | 192 | 203 | 357 | 361 | 220 | 220 |
| Ancud | ANCUD_46 | MLG43* | 302 | 318 | 197 | 201 | 261 | 261 | 192 | 203 | 357 | 357 | 222 | 222 |
| Ancud | ANCUD_47 | MLG66 | 316 | 316 | 197 | 197 | 261 | 261 | 203 | 203 | 357 | 357 | 222 | 222 |
| Ancud | ANCUD_51 | MLG73 | 316 | 316 | 197 | 197 | 300 | 300 | 201 | 201 | 357 | 357 | 222 | 222 |
| Ancud | ANCUD_53 | MLG05 | 302 | 302 | 197 | 197 | 261 | 261 | 201 | 203 | 357 | 357 | 222 | 222 |
| Ancud | ANCUD_54 | MLG03 | 302 | 302 | 197 | 197 | 261 | 261 | 201 | 201 | 357 | 357 | 222 | 222 |
| Ancud | ANCUD_55 | MLG60 | 316 | 316 | 197 | 197 | 261 | 261 | 201 | 201 | 357 | 357 | 220 | 220 |
| Ancud | ANCUD_56 | MLG48 | 302 | 318 | 197 | 201 | 261 | 297 | 192 | 201 | 357 | 361 | 222 | 222 |
| Ancud | ANCUD_57 | MLG26* | 302 | 316 | 197 | 197 | 261 | 297 | 201 | 203 | 357 | 361 | 222 | 222 |
| Ancud | ANCUD_58 | MLG48 | 302 | 318 | 197 | 201 | 261 | 297 | 192 | 201 | 357 | 361 | 222 | 222 |
| Ancud | ANCUD_59 | MLG06 | 302 | 302 | 197 | 197 | 261 | 261 | 203 | 203 | 357 | 357 | 222 | 222 |
| Ancud | ANCUD_60 | MLG06 | 302 | 302 | 197 | 197 | 261 | 261 | 203 | 203 | 357 | 357 | 222 | 222 |
| Ancud | ANCUD_61 | MLG48 | 302 | 318 | 197 | 201 | 261 | 297 | 192 | 201 | 357 | 361 | 222 | 222 |
| Ancud | ANCUD_62 | MLG06 | 302 | 302 | 197 | 197 | 261 | 261 | 203 | 203 | 357 | 357 | 222 | 222 |
| Ancud | ANCUD_63 | MLG39* | 302 | 318 | 197 | 197 | 261 | 297 | 201 | 203 | 357 | 357 | 222 | 222 |
| Ancud | ANCUD_64 | MLG33* | 302 | 316 | 197 | 197 | 300 | 300 | 192 | 201 | 357 | 357 | 222 | 222 |
| Ancud | ANCUD_65 | MLG17 | 302 | 302 | 197 | 197 | 300 | 300 | 203 | 203 | 357 | 357 | 222 | 222 |
| Ancud | ANCUD_67 | MLG03 | 302 | 302 | 197 | 197 | 261 | 261 | 201 | 201 | 357 | 357 | 222 | 222 |
| Ancud | ANCUD_68 | MLG38* | 302 | 316 | 197 | 201 | 261 | 297 | 201 | 203 | 357 | 357 | 222 | 222 |
| Ancud | ANCUD_69 | MLG63 | 316 | 316 | 197 | 197 | 261 | 261 | 201 | 203 | 357 | 357 | 220 | 220 |
| Ancud | ANCUD_70 | MLG94 | 318 | 318 | 201 | 201 | 261 | 261 | 201 | 201 | 357 | 357 | 222 | 222 |
| Ancud | ANCUD_71 | MLG18* | 302 | 302 | 197 | 201 | 261 | 300 | 201 | 201 | 357 | 357 | 222 | 222 |
| Ancud | ANCUD_72 | MLG18 | 302 | 302 | 197 | 201 | 261 | 300 | 201 | 201 | 357 | 357 | 222 | 222 |
| Ancud | ANCUD_73 | MLG58 | 304 | 316 | 197 | 197 | 261 | 261 | 201 | 201 | 357 | 357 | 222 | 222 |
| Ancud | ANCUD_74 | MLG08 | 302 | 302 | 197 | 197 | 261 | 300 | 201 | 201 | 357 | 357 | 222 | 222 |
| Ancud | ANCUD_75 | MLG61 | 316 | 316 | 197 | 197 | 261 | 261 | 201 | 201 | 357 | 357 | 220 | 222 |
| Ancud | ANCUD_76 | MLG16 | 302 | 302 | 197 | 197 | 300 | 300 | 203 | 203 | 357 | 357 | 220 | 220 |
| Ancud | ANCUD_77 | MLG75 | 316 | 316 | 197 | 197 | 300 | 300 | 203 | 203 | 357 | 357 | 220 | 220 |
| Ancud | ANCUD_78 | MLG27* | 302 | 316 | 197 | 197 | 261 | 300 | 201 | 201 | 357 | 357 | 220 | 220 |
| Ancud | ANCUD_79 | MLG21 | 302 | 316 | 197 | 197 | 261 | 261 | 192 | 203 | 357 | 361 | 222 | 222 |
| Ancud | ANCUD_80 | MLG03 | 302 | 302 | 197 | 197 | 261 | 261 | 201 | 201 | 357 | 357 | 222 | 222 |
| Ancud | ANCUD_81 | MLG28 | 302 | 316 | 197 | 197 | 261 | 300 | 201 | 201 | 357 | 357 | 222 | 222 |
| Ancud | ANCUD_84 | MLG48 | 302 | 318 | 197 | 201 | 261 | 297 | 192 | 201 | 357 | 361 | 222 | 222 |
| Ancud | ANCUD_85 | MLG04 | 302 | 302 | 197 | 197 | 261 | 261 | 201 | 203 | 357 | 357 | 220 | 220 |
| Ancud | ANCUD_87 | MLG37* | 302 | 316 | 197 | 197 | 300 | 300 | 203 | 205 | 357 | 357 | 222 | 222 |
| Ancud | ANCUD_88 | MLG05 | 302 | 302 | 197 | 197 | 261 | 261 | 201 | 203 | 357 | 357 | 222 | 222 |
| Ancud | ANCUD_90 | MLG06 | 302 | 302 | 197 | 197 | 261 | 261 | 203 | 203 | 357 | 357 | 222 | 222 |
| Ancud | ANCUD_91 | MLG02 | 302 | 302 | 197 | 197 | 261 | 261 | 201 | 201 | 357 | 357 | 220 | 220 |
| Ancud | ANCUD_92 | MLG74 | 316 | 316 | 197 | 197 | 300 | 300 | 201 | 203 | 357 | 357 | 222 | 222 |
| Ancud | ANCUD_93 | MLG24* | 302 | 316 | 197 | 197 | 261 | 263 | 201 | 201 | 357 | 357 | 222 | 222 |
| Ancud | ANCUD_94 | MLG48 | 302 | 318 | 197 | 201 | 261 | 297 | 192 | 201 | 357 | 361 | 222 | 222 |
| Ancud | ANCUD_95 | MLG64 | 316 | 316 | 197 | 197 | 261 | 261 | 201 | 203 | 357 | 357 | 222 | 222 |
| Ancud | ANCUD_97 | MLG08 | 302 | 302 | 197 | 197 | 261 | 300 | 201 | 201 | 357 | 357 | 222 | 222 |
| Ancud | ANCUD_98 | MLG72 | 316 | 316 | 197 | 197 | 263 | 263 | 201 | 201 | 357 | 357 | 222 | 222 |
| Ancud | ANCUD_99 | MLG34 | 302 | 316 | 197 | 197 | 300 | 300 | 201 | 201 | 357 | 357 | 220 | 220 |
| Ancud | ANCUD_100 | MLG71* | 316 | 316 | 197 | 197 | 261 | 300 | 203 | 203 | 357 | 357 | 222 | 224 |
| Ancud | ANCUD_101 | MLG40 | 302 | 318 | 197 | 197 | 261 | 297 | 201 | 203 | 357 | 361 | 220 | 220 |
| Ancud | ANCUD_102 | MLG40* | 302 | 318 | 197 | 197 | 261 | 297 | 201 | 203 | 357 | 361 | 220 | 220 |
| Ancud | ANCUD_103 | MLG15 | 302 | 302 | 197 | 197 | 300 | 300 | 201 | 203 | 357 | 357 | 222 | 222 |
| Ancud | ANCUD_105 | MLG02 | 302 | 302 | 197 | 197 | 261 | 261 | 201 | 201 | 357 | 357 | 220 | 220 |
| Ancud | ANCUD_106 | MLG31* | 302 | 316 | 197 | 197 | 261 | 300 | 203 | 203 | 357 | 357 | 222 | 222 |
| Ancud | ANCUD_107 | MLG21 | 302 | 316 | 197 | 197 | 261 | 261 | 192 | 203 | 357 | 361 | 222 | 222 |
| Ancud | ANCUD_108 | MLG60 | 316 | 316 | 197 | 197 | 261 | 261 | 201 | 201 | 357 | 357 | 220 | 220 |
| Ancud | ANCUD_109 | MLG10 | 302 | 302 | 197 | 197 | 261 | 300 | 203 | 203 | 357 | 357 | 222 | 222 |
| Ancud | ANCUD_110 | MLG14 | 302 | 302 | 197 | 197 | 300 | 300 | 201 | 201 | 357 | 357 | 222 | 222 |
| Ancud | ANCUD_111 | MLG70 | 316 | 316 | 197 | 197 | 261 | 300 | 201 | 203 | 357 | 357 | 222 | 222 |
| Ancud | ANCUD_112 | MLG62 | 316 | 316 | 197 | 197 | 261 | 261 | 201 | 201 | 357 | 357 | 222 | 222 |
| Ancud | ANCUD_113 | MLG62 | 316 | 316 | 197 | 197 | 261 | 261 | 201 | 201 | 357 | 357 | 222 | 222 |
| Ancud | ANCUD_116 | MLG14 | 302 | 302 | 197 | 197 | 300 | 300 | 201 | 201 | 357 | 357 | 222 | 222 |
| Ancud | ANCUD_117 | MLG09* | 302 | 302 | 197 | 197 | 261 | 300 | 201 | 203 | 357 | 357 | 222 | 222 |
| Ancud | ANCUD_118 | MLG32* | 302 | 316 | 197 | 197 | 261 | 300 | 203 | 205 | 357 | 357 | 220 | 220 |
| Ancud | ANCUD_120 | MLG80 | 316 | 318 | 197 | 197 | 297 | 300 | 192 | 201 | 357 | 361 | 222 | 222 |
| Ancud | ANCUD_122 | MLG30 | 302 | 316 | 197 | 197 | 261 | 300 | 201 | 203 | 357 | 357 | 222 | 222 |
| Ancud | ANCUD_123 | MLG80 | 316 | 318 | 197 | 197 | 297 | 300 | 192 | 201 | 357 | 361 | 222 | 222 |
| Ancud | ANCUD_125 | MLG47 | 302 | 318 | 197 | 201 | 261 | 297 | 192 | 201 | 357 | 361 | 220 | 220 |
| Ancud | ANCUD_126 | MLG80* | 316 | 318 | 197 | 197 | 297 | 300 | 192 | 201 | 357 | 361 | 222 | 222 |
| Ancud | ANCUD_127 | MLG30 | 302 | 316 | 197 | 197 | 261 | 300 | 201 | 203 | 357 | 357 | 222 | 222 |
| Ancud | ANCUD_128 | MLG30 | 302 | 316 | 197 | 197 | 261 | 300 | 201 | 203 | 357 | 357 | 222 | 222 |
| Ancud | ANCUD_129 | MLG55 | 302 | 318 | 197 | 201 | 297 | 300 | 192 | 201 | 357 | 361 | 220 | 220 |
| Ancud | ANCUD_130 | MLG30* | 302 | 316 | 197 | 197 | 261 | 300 | 201 | 203 | 357 | 357 | 222 | 222 |
| Chaica | CHAICA_01 | MLG83 | 316 | 318 | 201 | 201 | 297 | 297 | 192 | 192 | 361 | 361 | 220 | 220 |
| Chaica | CHAICA_02 | MLG84 | 318 | 318 | 197 | 197 | 261 | 261 | 192 | 203 | 357 | 357 | 220 | 220 |
| Chaica | CHAICA_03 | MLG88* | 318 | 318 | 197 | 197 | 297 | 297 | 192 | 203 | 357 | 361 | 220 | 220 |
| Chaica | CHAICA_04 | MLG97 | 318 | 318 | 201 | 201 | 297 | 297 | 192 | 192 | 357 | 361 | 220 | 220 |
| Chaica | CHAICA_05 | MLG30 | 302 | 316 | 197 | 197 | 261 | 300 | 201 | 203 | 357 | 357 | 222 | 222 |
| Chaica | CHAICA_06 | MLG80 | 316 | 318 | 197 | 197 | 297 | 300 | 192 | 201 | 357 | 361 | 222 | 222 |
| Chaica | CHAICA_07 | MLG56 | 302 | 318 | 197 | 201 | 297 | 300 | 192 | 203 | 357 | 361 | 220 | 220 |
| Chaica | CHAICA_08 | MLG50 | 302 | 318 | 197 | 201 | 261 | 297 | 192 | 203 | 357 | 361 | 220 | 220 |
| Chaica | CHAICA_09 | MLG90* | 318 | 318 | 197 | 197 | 297 | 300 | 192 | 203 | 357 | 361 | 220 | 220 |
| Chaica | CHAICA_10 | MLG55 | 302 | 318 | 197 | 201 | 297 | 300 | 192 | 201 | 357 | 361 | 220 | 220 |
| Chaica | CHAICA_11 | MLG50 | 302 | 318 | 197 | 201 | 261 | 297 | 192 | 203 | 357 | 361 | 220 | 220 |
| Chaica | CHAICA_12 | MLG79 | 316 | 318 | 197 | 197 | 297 | 297 | 192 | 201 | 357 | 361 | 222 | 222 |
| Chaica | CHAICA_13 | MLG50 | 302 | 318 | 197 | 201 | 261 | 297 | 192 | 203 | 357 | 361 | 220 | 220 |
| Chaica | CHAICA_14 | MLG96 | 318 | 318 | 201 | 201 | 297 | 297 | 192 | 192 | 357 | 357 | 220 | 220 |
| Chaica | CHAICA_15 | MLG80 | 316 | 318 | 197 | 197 | 297 | 300 | 192 | 201 | 357 | 361 | 222 | 222 |
| Chaica | CHAICA_16 | MLG41* | 302 | 318 | 197 | 197 | 261 | 300 | 201 | 203 | 357 | 357 | 222 | 222 |
| Chaica | CHAICA_17 | MLG96 | 318 | 318 | 201 | 201 | 297 | 297 | 192 | 192 | 357 | 357 | 220 | 220 |
| Chaica | CHAICA_18 | MLG80 | 316 | 318 | 197 | 197 | 297 | 300 | 192 | 201 | 357 | 361 | 222 | 222 |
| Chaica | CHAICA_19 | MLG79 | 316 | 318 | 197 | 197 | 297 | 297 | 192 | 201 | 357 | 361 | 222 | 222 |
| Chaica | CHAICA_20 | MLG79 | 316 | 318 | 197 | 197 | 297 | 297 | 192 | 201 | 357 | 361 | 222 | 222 |
| Chaica | CHAICA_21 | MLG30 | 302 | 316 | 197 | 197 | 261 | 300 | 201 | 203 | 357 | 357 | 222 | 222 |
| Chaica | CHAICA_22 | MLG44* | 302 | 318 | 197 | 201 | 261 | 261 | 192 | 203 | 357 | 361 | 220 | 220 |
| Chaica | CHAICA_23 | MLG79 | 316 | 318 | 197 | 197 | 297 | 297 | 192 | 201 | 357 | 361 | 222 | 222 |
| Chaica | CHAICA_24 | MLG87 | 318 | 318 | 197 | 197 | 297 | 297 | 192 | 201 | 357 | 361 | 222 | 222 |
| Chaica | CHAICA_25 | MLG89 | 318 | 318 | 197 | 197 | 297 | 300 | 192 | 201 | 357 | 361 | 222 | 222 |
| Chaica | CHAICA_26 | MLG87* | 318 | 318 | 197 | 197 | 297 | 297 | 192 | 201 | 357 | 361 | 222 | 222 |
| Chaica | CHAICA_27 | MLG30 | 302 | 316 | 197 | 197 | 261 | 300 | 201 | 203 | 357 | 357 | 222 | 222 |
| Chaica | CHAICA_28 | MLG89* | 318 | 318 | 197 | 197 | 297 | 300 | 192 | 201 | 357 | 361 | 222 | 222 |
| Chaica | CHAICA_29 | MLG01 | 302 | 302 | 197 | 197 | 261 | 261 | 197 | 201 | 357 | 357 | 222 | 222 |
| Chaica | CHAICA_30 | MLG30 | 302 | 316 | 197 | 197 | 261 | 300 | 201 | 203 | 357 | 357 | 222 | 222 |
| Chaica | CHAICA_31 | MLG96 | 318 | 318 | 201 | 201 | 297 | 297 | 192 | 192 | 357 | 357 | 220 | 220 |
| Chaica | CHAICA_32 | MLG96 | 318 | 318 | 201 | 201 | 297 | 297 | 192 | 192 | 357 | 357 | 220 | 220 |
| Chaica | CHAICA_33 | MLG08 | 302 | 302 | 197 | 197 | 261 | 300 | 201 | 201 | 357 | 357 | 222 | 222 |
| Chaica | CHAICA_34 | MLG50 | 302 | 318 | 197 | 201 | 261 | 297 | 192 | 203 | 357 | 361 | 220 | 220 |
| Chaica | CHAICA_35 | MLG22* | 302 | 316 | 197 | 197 | 261 | 261 | 201 | 203 | 357 | 357 | 222 | 222 |
| Chaica | CHAICA_36 | MLG30 | 302 | 316 | 197 | 197 | 261 | 300 | 201 | 203 | 357 | 357 | 222 | 222 |
| Chaica | CHAICA_37 | MLG70 | 316 | 316 | 197 | 197 | 261 | 300 | 201 | 203 | 357 | 357 | 222 | 222 |
| Chaica | CHAICA_38 | MLG50 | 302 | 318 | 197 | 201 | 261 | 297 | 192 | 203 | 357 | 361 | 220 | 220 |
| Chaica | CHAICA_39 | MLG80 | 316 | 318 | 197 | 197 | 297 | 300 | 192 | 201 | 357 | 361 | 222 | 222 |
| Chaica | CHAICA_40 | MLG80 | 316 | 318 | 197 | 197 | 297 | 300 | 192 | 201 | 357 | 361 | 222 | 222 |
| Chaica | CHAICA_42 | MLG97 | 318 | 318 | 201 | 201 | 297 | 297 | 192 | 192 | 357 | 361 | 220 | 220 |
| Chaica | CHAICA_43 | MLG98* | 318 | 318 | 201 | 201 | 297 | 300 | 192 | 192 | 357 | 361 | 220 | 220 |
| Chaica | CHAICA_44 | MLG50 | 302 | 318 | 197 | 201 | 261 | 297 | 192 | 203 | 357 | 361 | 220 | 220 |
| Chaica | CHAICA_45 | MLG49* | 302 | 318 | 197 | 201 | 261 | 297 | 192 | 203 | 357 | 357 | 220 | 220 |
| Chaica | CHAICA_46 | MLG19 | 302 | 302 | 197 | 201 | 297 | 297 | 192 | 192 | 361 | 361 | 220 | 220 |
| Chaica | CHAICA_47 | MLG30 | 302 | 316 | 197 | 197 | 261 | 300 | 201 | 203 | 357 | 357 | 222 | 222 |
| Chaica | CHAICA_48 | MLG83 | 316 | 318 | 201 | 201 | 297 | 297 | 192 | 192 | 361 | 361 | 220 | 220 |
| Chaica | CHAICA_49 | MLG83 | 316 | 318 | 201 | 201 | 297 | 297 | 192 | 192 | 361 | 361 | 220 | 220 |
| Chaica | CHAICA_50 | MLG83 | 316 | 318 | 201 | 201 | 297 | 297 | 192 | 192 | 361 | 361 | 220 | 220 |
| Chaica | CHAICA_51 | MLG83 | 316 | 318 | 201 | 201 | 297 | 297 | 192 | 192 | 361 | 361 | 220 | 220 |
| Chaica | CHAICA_52 | MLG30 | 302 | 316 | 197 | 197 | 261 | 300 | 201 | 203 | 357 | 357 | 222 | 222 |
| Chaica | CHAICA_53 | MLG30 | 302 | 316 | 197 | 197 | 261 | 300 | 201 | 203 | 357 | 357 | 222 | 222 |
| Chaica | CHAICA_55 | MLG30 | 302 | 316 | 197 | 197 | 261 | 300 | 201 | 203 | 357 | 357 | 222 | 222 |
| Chaica | CHAICA_56 | MLG30 | 302 | 316 | 197 | 197 | 261 | 300 | 201 | 203 | 357 | 357 | 222 | 222 |
| Chaica | CHAICA_57 | MLG30 | 302 | 316 | 197 | 197 | 261 | 300 | 201 | 203 | 357 | 357 | 222 | 222 |
| Chaica | CHAICA_59 | MLG50 | 302 | 318 | 197 | 201 | 261 | 297 | 192 | 203 | 357 | 361 | 220 | 220 |
| Chaica | CHAICA_60 | MLG30 | 302 | 316 | 197 | 197 | 261 | 300 | 201 | 203 | 357 | 357 | 222 | 222 |
| Chaica | CHAICA_61 | MLG50 | 302 | 318 | 197 | 201 | 261 | 297 | 192 | 203 | 357 | 361 | 220 | 220 |
| Chaica | CHAICA_62 | MLG57* | 302 | 318 | 201 | 201 | 261 | 300 | 192 | 201 | 357 | 361 | 222 | 222 |
| Chaica | CHAICA_63 | MLG96 | 318 | 318 | 201 | 201 | 297 | 297 | 192 | 192 | 357 | 357 | 220 | 220 |
| Chaica | CHAICA_64 | MLG30 | 302 | 316 | 197 | 197 | 261 | 300 | 201 | 203 | 357 | 357 | 222 | 222 |
| Chaica | CHAICA_65 | MLG96 | 318 | 318 | 201 | 201 | 297 | 297 | 192 | 192 | 357 | 357 | 220 | 220 |
| Chaica | CHAICA_66 | MLG96 | 318 | 318 | 201 | 201 | 297 | 297 | 192 | 192 | 357 | 357 | 220 | 220 |
| Chaica | CHAICA_67 | MLG96 | 318 | 318 | 201 | 201 | 297 | 297 | 192 | 192 | 357 | 357 | 220 | 220 |
| Chaica | CHAICA_68 | MLG54 | 302 | 318 | 197 | 201 | 297 | 297 | 192 | 203 | 357 | 361 | 220 | 220 |
| Chaica | CHAICA_69 | MLG96 | 318 | 318 | 201 | 201 | 297 | 297 | 192 | 192 | 357 | 357 | 220 | 220 |
| Chaica | CHAICA_70 | MLG96 | 318 | 318 | 201 | 201 | 297 | 297 | 192 | 192 | 357 | 357 | 220 | 220 |
| Chaica | CHAICA_71 | MLG96 | 318 | 318 | 201 | 201 | 297 | 297 | 192 | 192 | 357 | 357 | 220 | 220 |
| Chaica | CHAICA_72 | MLG96 | 318 | 318 | 201 | 201 | 297 | 297 | 192 | 192 | 357 | 357 | 220 | 220 |
| Chaica | CHAICA_73 | MLG80 | 316 | 318 | 197 | 197 | 297 | 300 | 192 | 201 | 357 | 361 | 222 | 222 |
| Chaica | CHAICA_74 | MLG09* | 302 | 302 | 197 | 197 | 261 | 300 | 201 | 203 | 357 | 357 | 222 | 222 |
| Chaica | CHAICA_75 | MLG91* | 318 | 318 | 197 | 201 | 261 | 261 | 192 | 201 | 357 | 361 | 220 | 220 |
| Chaica | CHAICA_76 | MLG79 | 316 | 318 | 197 | 197 | 297 | 297 | 192 | 201 | 357 | 361 | 222 | 222 |
| Chaica | CHAICA_77 | MLG09 | 302 | 302 | 197 | 197 | 261 | 300 | 201 | 203 | 357 | 357 | 222 | 222 |
| Chaica | CHAICA_78 | MLG55 | 302 | 318 | 197 | 201 | 297 | 300 | 192 | 201 | 357 | 361 | 220 | 220 |
| Chaica | CHAICA_79 | MLG30 | 302 | 316 | 197 | 197 | 261 | 300 | 201 | 203 | 357 | 357 | 222 | 222 |
| Chaica | CHAICA_80 | MLG96 | 318 | 318 | 201 | 201 | 297 | 297 | 192 | 192 | 357 | 357 | 220 | 220 |
| Chaica | CHAICA_81 | MLG95* | 318 | 318 | 201 | 201 | 261 | 297 | 192 | 192 | 357 | 357 | 220 | 222 |
| Chaica | CHAICA_82 | MLG96 | 318 | 318 | 201 | 201 | 297 | 297 | 192 | 192 | 357 | 357 | 220 | 220 |
| Chaica | CHAICA_83 | MLG88 | 318 | 318 | 197 | 197 | 297 | 297 | 192 | 203 | 357 | 361 | 220 | 220 |
| Chaica | CHAICA_84 | MLG80 | 316 | 318 | 197 | 197 | 297 | 300 | 192 | 201 | 357 | 361 | 222 | 222 |
| Chaica | CHAICA_85 | MLG80 | 316 | 318 | 197 | 197 | 297 | 300 | 192 | 201 | 357 | 361 | 222 | 222 |
| Chaica | CHAICA_86 | MLG30 | 302 | 316 | 197 | 197 | 261 | 300 | 201 | 203 | 357 | 357 | 222 | 222 |
| Chaica | CHAICA_87 | MLG54* | 302 | 318 | 197 | 201 | 297 | 297 | 192 | 203 | 357 | 361 | 220 | 220 |
| Chaica | CHAICA_88 | MLG80 | 316 | 318 | 197 | 197 | 297 | 300 | 192 | 201 | 357 | 361 | 222 | 222 |
| Chaica | CHAICA_89 | MLG30 | 302 | 316 | 197 | 197 | 261 | 300 | 201 | 203 | 357 | 357 | 222 | 222 |
| Chaica | CHAICA_90 | MLG30 | 302 | 316 | 197 | 197 | 261 | 300 | 201 | 203 | 357 | 357 | 222 | 222 |
| Chaica | CHAICA_91 | MLG30 | 302 | 316 | 197 | 197 | 261 | 300 | 201 | 203 | 357 | 357 | 222 | 222 |
| Chaica | CHAICA_92 | MLG79 | 316 | 318 | 197 | 197 | 297 | 297 | 192 | 201 | 357 | 361 | 222 | 222 |
| Chaica | CHAICA_93 | MLG80* | 316 | 318 | 197 | 197 | 297 | 300 | 192 | 201 | 357 | 361 | 222 | 222 |
| Chaica | CHAICA_94 | MLG74 | 316 | 316 | 197 | 197 | 300 | 300 | 201 | 203 | 357 | 357 | 222 | 222 |
| Chaica | CHAICA_95 | MLG96 | 318 | 318 | 201 | 201 | 297 | 297 | 192 | 192 | 357 | 357 | 220 | 220 |
| Chaica | CHAICA_96 | MLG53 | 302 | 318 | 197 | 201 | 297 | 297 | 192 | 201 | 357 | 361 | 220 | 220 |
| Chaica | CHAICA_97 | MLG79* | 316 | 318 | 197 | 197 | 297 | 297 | 192 | 201 | 357 | 361 | 222 | 222 |
| Chaica | CHAICA_98 | MLG30 | 302 | 316 | 197 | 197 | 261 | 300 | 201 | 203 | 357 | 357 | 222 | 222 |
| Chaica | CHAICA_99 | MLG20* | 302 | 316 | 197 | 197 | 261 | 261 | 192 | 201 | 357 | 361 | 222 | 222 |
| Chaica | CHAICA_100 | MLG80 | 316 | 318 | 197 | 197 | 297 | 300 | 192 | 201 | 357 | 361 | 222 | 222 |
| Chaica | CHAICA_101 | MLG30* | 302 | 316 | 197 | 197 | 261 | 300 | 201 | 203 | 357 | 357 | 222 | 222 |
| Chaica | CHAICA_102 | MLG80 | 316 | 318 | 197 | 197 | 297 | 300 | 192 | 201 | 357 | 361 | 222 | 222 |
| Chaica | CHAICA_103 | MLG30 | 302 | 316 | 197 | 197 | 261 | 300 | 201 | 203 | 357 | 357 | 222 | 222 |
| Chaica | CHAICA_104 | MLG55 | 302 | 318 | 197 | 201 | 297 | 300 | 192 | 201 | 357 | 361 | 220 | 220 |
| Chaica | CHAICA_105 | MLG80 | 316 | 318 | 197 | 197 | 297 | 300 | 192 | 201 | 357 | 361 | 222 | 222 |
| Chaica | CHAICA_106 | MLG80 | 316 | 318 | 197 | 197 | 297 | 300 | 192 | 201 | 357 | 361 | 222 | 222 |
| Chaica | CHAICA_107 | MLG46* | 302 | 318 | 197 | 201 | 261 | 297 | 192 | 192 | 361 | 361 | 222 | 222 |
| Chaica | CHAICA_108 | MLG96 | 318 | 318 | 201 | 201 | 297 | 297 | 192 | 192 | 357 | 357 | 220 | 220 |
| Chaica | CHAICA_109 | MLG77 | 316 | 316 | 201 | 201 | 297 | 297 | 192 | 192 | 361 | 361 | 220 | 220 |
| Chaica | CHAICA_111 | MLG79 | 316 | 318 | 197 | 197 | 297 | 297 | 192 | 201 | 357 | 361 | 222 | 222 |
| Chaica | CHAICA_112 | MLG80 | 316 | 318 | 197 | 197 | 297 | 300 | 192 | 201 | 357 | 361 | 222 | 222 |
| Chaica | CHAICA_113 | MLG82* | 316 | 318 | 197 | 201 | 300 | 300 | 192 | 192 | 357 | 361 | 220 | 220 |
| Chaica | CHAICA_114 | MLG86 | 318 | 318 | 197 | 197 | 297 | 297 | 192 | 192 | 361 | 361 | 220 | 220 |
| Chaica | CHAICA_115 | MLG80 | 316 | 318 | 197 | 197 | 297 | 300 | 192 | 201 | 357 | 361 | 222 | 222 |
| Chaica | CHAICA_116 | MLG74 | 316 | 316 | 197 | 197 | 300 | 300 | 201 | 203 | 357 | 357 | 222 | 222 |
| Chaica | CHAICA_117 | MLG79 | 316 | 318 | 197 | 197 | 297 | 297 | 192 | 201 | 357 | 361 | 222 | 222 |
| Chaica | CHAICA_118 | MLG34 | 302 | 316 | 197 | 197 | 300 | 300 | 201 | 201 | 357 | 357 | 220 | 220 |
| Chaica | CHAICA_119 | MLG70* | 316 | 316 | 197 | 197 | 261 | 300 | 201 | 203 | 357 | 357 | 222 | 222 |
| Chaica | CHAICA_120 | MLG79 | 316 | 318 | 197 | 197 | 297 | 297 | 192 | 201 | 357 | 361 | 222 | 222 |
| Chaica | CHAICA_121 | MLG73 | 316 | 316 | 197 | 197 | 300 | 300 | 201 | 201 | 357 | 357 | 222 | 222 |
| Chaica | CHAICA_122 | MLG08 | 302 | 302 | 197 | 197 | 261 | 300 | 201 | 201 | 357 | 357 | 222 | 222 |
| Chaica | CHAICA_123 | MLG39* | 302 | 318 | 197 | 197 | 261 | 297 | 201 | 203 | 357 | 357 | 222 | 222 |
| Chaica | CHAICA_124 | MLG51 | 302 | 318 | 197 | 201 | 261 | 297 | 192 | 203 | 357 | 361 | 222 | 222 |
| Chaica | CHAICA_125 | MLG53* | 302 | 318 | 197 | 201 | 297 | 297 | 192 | 201 | 357 | 361 | 220 | 220 |
| Chaica | CHAICA_126 | MLG26* | 302 | 316 | 197 | 197 | 261 | 297 | 201 | 203 | 357 | 361 | 222 | 222 |
| Chaica | CHAICA_127 | MLG43* | 302 | 318 | 197 | 201 | 261 | 261 | 192 | 203 | 357 | 357 | 222 | 222 |
| Chaica | CHAICA_128 | MLG30 | 302 | 316 | 197 | 197 | 261 | 300 | 201 | 203 | 357 | 357 | 222 | 222 |
| Chaica | CHAICA_129 | MLG60 | 316 | 316 | 197 | 197 | 261 | 261 | 201 | 201 | 357 | 357 | 220 | 220 |
| Chaica | CHAICA_130 | MLG76 | 316 | 316 | 197 | 197 | 300 | 300 | 203 | 203 | 357 | 357 | 222 | 222 |

**Supplementary Table 2:** Change in specific growth rate of each 32 *Agarophyton chilense* genotypes (16 from locality of Ancud and 16 from the locality of Chaica) grown in two distinct genotypic diversity treatments: 1-genotype and 4-genotypes. Specific growth rate (SGR) was measured as % wet weight · day^-1^. Significant differences between distinct genotypic diversity treatments was tested using 1-way ANOVA independently for each locality of origin; values of p < 0.05 noted in bold. Trend of SGR variation between 1-genotype and 4-genotypes treatments are represented by arrows.

| **Locality of origin** | **Genotype identity** | **1-genotype treatment** | **Trend of SGR variation** | **p-value** | **4-genotypes treatment** |
| --- | --- | --- | --- | --- | --- |
| Ancud | MLG09 | 3.463 ± 1.063 | → | 0.337 | 4.198 ± 1.456 |
|  | MLG18 | 5.449 ± 1.627 | ↘ | **0.0071** | 2.942 ± 1.956 |
|  | MLG21 | 6.286 ± 1.042 | ↘ | **0.0294** | 4.347 ± 1.033 |
|  | MLG25 | 6.624 ± 0.901 | ↘ | **<0.0001** | 2.135 ± 0.638 |
|  | MLG28 | 2.744 ± 0.549 | ↗ | **0.0335** | 4.631 ± 1.771 |
|  | MLG30 | 4.573 ± 1.612 | ↘ | **0.0010** | 1.771 ± 1.144 |
|  | MLG36 | 6.879 ± 2.595 | → | 0.0718 | 4.475 ± 2.398 |
|  | MLG39 | 4.880 ± 1.466 | → | 0.3914 | 4.252 ± 1.911 |
|  | MLG40 | 7.083 ± 1.587 | ↘ | **0.0067** | 4.418 ± 2.807 |
|  | MLG43 | 8.679 ± 0.046 | ↘ | **0.0011** | 3.954 ± 2.670 |
|  | MLG68 | 2.891 ± 2.135 | → | 0.6348 | 3.256 ± 0.684 |
|  | MLG69 | 5.987 ± 2.182 | ↘ | **0.0100** | 4.221 ± 0.492 |
|  | MLG70 | 2.620 ± 1.837 | → | 0.1244 | 4.127 ± 2.202 |
|  | MLG80 | 6.889 ± 1.553 | ↘ | **<0.0001** | 3.442 ± 1.773 |
|  | MLG92 | 6.118 ± 1.012 | ↘ | **0.0054** | 3.315 ± 1.300 |
|  | MLG93 | 5.972 ± 0.985 | ↘ | **<0.0001** | 1.914 ± 0.900 |
| Chaica | MLG09 | 6.070 ± 2.004 | ↘ | **0.036** | 4.593 ± 1.998 |
|  | MLG20 | 4.937 ± 2.139 | → | 0.180 | 3.516 ± 2.587 |
|  | MLG30 | 4.970 ± 2.712 | → | 0.078 | 6.145 ± 1.543 |
|  | MLG41 | 4.886 ± 1.433 | → | 0.392 | 4.234 ± 2.190 |
|  | MLG43 | 5.286 ± 3.008 | → | 0.104 | 3.190 ± 1.385 |
|  | MLG44 | 5.065 ± 1.431 | → | 0.451 | 4.297 ± 1.972 |
|  | MLG53 | 3.310 ± 1.206 | → | 0.241 | 2.151 ± 0.258 |
|  | MLG54 | 6.532 ± 2.505 | ↘ | **<0.0001** | 2.258 ± 0.884 |
|  | MLG70 | 3.523 ± 1.160 | → | 0.065 | 1.488 ± 0.739 |
|  | MLG79 | 2.561 ± 2.529 | → | 0.093 | 4.277 ± 2.577 |
|  | MLG80 | 6.170 ± 1.616 | ↘ | **0.033** | 4.241 ± 2.115 |
|  | MLG87 | 5.585 ± 1.601 | ↘ | **0.033** | 3.422 ± 1.389 |
|  | MLG88 | 3.131 ± 0.141 | → | 0.937 | 3.208 ± 2.061 |
|  | MLG89 | 3.893 ± 2.103 | → | 0.833 | 4.017 ± 1.954 |
|  | MLG90 | 1.555 ± 1.335 | ↗ | **0.001** | 4.430 ± 1.919 |
|  | MLG91 | 4.410 ± 1.791 | → | 0.306 | 3.837 ± 1.223 |
|  | MLG30 | 4.970 ± 2.712 | → | 0.078 | 6.145 ± 1.543 |
|  | MLG41 | 4.886 ± 1.433 | → | 0.392 | 4.234 ± 2.190 |
|  | MLG43 | 5.286 ± 3.008 | → | 0.104 | 3.190 ± 1.385 |
|  | MLG44 | 5.065 ± 1.431 | → | 0.451 | 4.297 ± 1.972 |
|  | MLG53 | 3.310 ± 1.206 | → | 0.241 | 2.151 ± 0.258 |
